# Comparative Analysis of the Effects of Mogroside V, Epigallocatechin Gallate, and Resveratrol on Growth Performance, Blood Parameters, Rumen Microbiota, and Short‐Chain Fatty Acid Metabolism in Heat‐Stressed Hu Sheep

**DOI:** 10.1002/fsn3.71455

**Published:** 2026-01-15

**Authors:** Yirong Wei, Jun Lu, Shaoqiang Wu, Zhihua Mo, Haien He, Yulong Shen, Jianwei Zou, Cheng Xing, Yanna Huang, Qinyang Jiang

**Affiliations:** ^1^ College of Animal Science and Technology Guangxi University Nanning Guangxi China; ^2^ Guangxi Key Laboratory of Animal Breeding, Disease Control and Prevention Guangxi University Nanning Guangxi China

**Keywords:** growth performance, heat stress, Hu sheep, microbiota, natural plant extracts, short‐chain fatty acids

## Abstract

Hu sheep are highly prized for their tender meat, but heat stress (HS) caused by the high temperature and humidity in southern China severely impacts their performance. This study compared the alleviating effects of Mogroside V (Mog V), epigallocatechin gallate (EGCG), and resveratrol (RES) on HS in Hu sheep. Forty male Hu sheep were randomly divided into a control group and three treatment groups (*n* = 10), each with a pen. The sheep were housed under HS for 60 days. Body weight, feed conversion ratio (FCR), respiratory rate (RR), and rectal temperature (RT) were monitored. Blood physiological parameters, HSPs, antioxidant enzymes, and inflammatory factors were measured. 16S rRNA sequencing and targeted metabolomics were used to analyze the correlation between rumen microbiota and short‐chain fatty acid (SCFA) metabolites. Results showed that all three extracts significantly increased final weight, total weight gain, and daily weight gain, while reducing FCR, RR, and RT. They also decreased HSP70/90, MDA, and the inflammatory factors TNF‐α, IL‐1β, and IL‐6, and increased antioxidant enzyme activity. Microbiome and metabolome analysis revealed that RES increased *Verrucomicrobia* and *Fibrobacterium*, promoting propionic and butyric acid production; Mog V enriched Firmicutes and Clostridium succinate, promoting energy metabolism; and EGCG regulated acetate metabolism through Lactobacilli, inhibiting pathogenic bacteria. In summary, all three plant extracts alleviated the physiological damage caused by HS and improved production performance, with Mog V showing the most significant effect and possessing high potential for application.

## Graphical abstract

Comparative effects of Mogroside V, EGCG, and resveratrol in heat‐stressed Hu sheep.

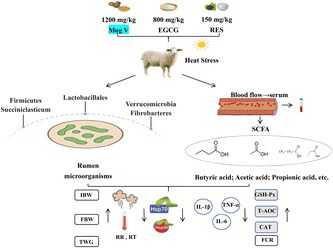

## 1 Introduction

Hu sheep have been designated as a nationally protected indigenous breed in China (Zhang et al. 2024) and are noted for early maturity, high reproductive performance, tolerance to heat and humidity, high lambing rates, and superior meat quality. In subtropical regions such as Guangxi, the use of agricultural by‐products and intensive farming systems has made Hu sheep a predominant breed in southern China. However, under the hot and humid conditions typical of southern summers, intensively reared Hu sheep are susceptible to heat stress (HS), which adversely affects productivity and health. HS is a physiological response to elevated temperature and humidity that impairs thermoregulation, leading to diminished antioxidant capacity, suppressed immune function, and reduced feed conversion efficiency, thereby compromising growth performance, intestinal microbial balance, and overall health (Elbaz et al. 2022; Hatipoglu et al. 2024; Yu et al. 2020).

HS profoundly impairs growth and health in Hu sheep, primarily reflected in reduced feed intake, inadequate energy intake, and metabolic disorders (Chauhan et al. 2023; Vicente Pérez et al. 2020). Under environmental conditions of 33°C–45°C and Temperature‐Humidity Index (THI) > 80, the feed intake of HS‐exposed lambs decreased by ~17.5%. Notably, even when feed intake was comparable to that of controls, daily weight gain was significantly reduced, indicating that HS not only suppresses feeding behavior but also disrupts metabolic processes, thereby reducing growth efficiency (Xin et al. 2018). Pragna et al. (2018) reported that HS increases maintenance energy requirements by 10%–32% (Tüfekci and Sejian 2023) and suppresses the activity of *fibrolytic* bacteria such as *Fibrobacter* in the rumen, thus reducing energy utilization efficiency (Pragna et al. 2018).

At the physiological level, HS markedly increases respiratory rate (RR) and rectal temperature (RT) in animals, with significant breed‐dependent variation in tolerance (Romero et al. 2013; Sejian et al. 2019; Srikandakumar et al. 2003). Although an elevated RR facilitates heat dissipation, excessive increases can lead to respiratory alkalosis and oxidative damage (Fischer et al. 2017; Nagayach et al. 2017). HS also disrupts the oxidative–antioxidative balance by inhibiting antioxidant enzymes, including superoxide dismutase (SOD) and glutathione peroxidase (GSH‐Px), resulting in elevated malondialdehyde (MDA) levels and reduced total antioxidant capacity (T‐AOC), thereby aggravating lipid peroxidation (Liu et al. 2022).

With respect to immunity, HS suppresses humoral immune markers, namely IgG, IgA, and IgM, while simultaneously inducing pro‐inflammatory cytokines such as TNF‐α and IL‐1β and impairing T‐cell function (Park et al. 2021). Shi et al. (2020) reported that, using a 28‐day HS model, pro‐inflammatory cytokines significantly increased from day 14 onward, whereas IL‐2 declined by day 28, indicating immune suppression.

HS further disrupts rumen microbial ecology by lowering pH and increasing temperature, reducing *fibrolytic* bacteria such as *Ruminococcaceae* while promoting lactate‐producing bacteria such as *Prevotella*, thereby increasing the risk of acidosis. Park et al. (2022) reported that HS increases the relative abundance of Bacteroidetes while reducing Firmicutes, resulting in structural and functional remodeling of the rumen microbiota. Kim et al. (2022) and Baek et al. (2020) observed that HS decreases acetate production and increases lactate levels, thereby altering metabolic pathways. In contrast, Zhang et al. (2024) demonstrated that HS increases the abundance of Firmicutes while decreasing Bacteroidetes, enhances AMPK and nitrogen metabolism pathways while suppressing lipid metabolism, and showed that specific bacterial taxa correlate with HSP70 expression, thereby suggesting potential roles in heat tolerance regulation.

Collectively, HS impairs Hu sheep productivity through multiple pathways involving metabolism, immunity, and rumen microbial ecology, highlighting the need for molecular‐level investigations to inform heat‐tolerant breeding strategies. Owing to their safety and multifunctionality, natural plant extracts have been shown to mitigate HS in livestock (Manuelian et al. 2021). Among them, Mogroside V (Mog V), Epigallocatechin Gallate (EGCG), and Resveratrol (RES) can effectively increase the activity of antioxidant enzymes such as SOD and GSH‐Px, reduce MDA levels, thereby alleviating oxidative damage caused by HS (Chen et al. 2007; Isbrucker et al. 2006; Li et al. 2019; Meng et al. 2020; Smith et al. 2013). In addition, they can inhibit key inflammatory signaling pathways (e.g., NF‐κB), suppress the release of pro‐inflammatory cytokines, and improve systemic inflammatory status (Li et al. 2019; Liu et al. 2016; Menegazzi et al. 2020). These compounds also directly or indirectly regulate the expression of HSP70 and exert protective effects in multiple organs by regulating apoptosis and metabolic pathways (e.g., AMPK/SIRT1), thereby maintaining the stability of intestinal, hepatic, and immune functions (Liu et al. 2014; Lu et al. 2023; Orhan et al. 2013).

This study evaluated the comparative effects of natural plant extracts (Mog V, EGCG, and RES) on growth performance, blood antioxidant and inflammatory parameters, rumen microbiota, and metabolite profiles in Hu sheep under heat stress to assess their potential in mitigating HS, enhancing productivity, and modulating rumen microbial ecology.

## 2 Materials and Methods

The animal experiment was reviewed and approved by the Animal Ethics Committee of Guangxi University (Protocol No. Gxu‐20230135). The Hu sheep were obtained from Guangxi Anxin Animal Husbandry Co. Ltd., and the feeding trial was carried out at its fattening farm in Dahua Yao Autonomous County, Hechi City, Guangxi, China. The additives included Mog V (≥ 90% purity; Shandong Fuwangjia Biotechnology Co. Ltd., China), EGCG (≥ 98% purity; Wuhan Costain Biotechnology Co. Ltd., China), and RES (≥ 99% purity; Shaanxi Ciyuan Biotechnology Co. Ltd., China).

### 2.1 Animals and Experimental Diets

Our research group has previously systematically evaluated Mog V, EGCG, and RES across multiple animal models, including Hu sheep, goats, and fattening pigs, with particular emphasis on meat quality traits, intramuscular fat (IMF) deposition, and related physiological parameters. These studies provide a robust experimental basis for the selection of antioxidant types and the determination of appropriate dietary inclusion levels. Previous studies have shown that dietary supplementation with RES (150 mg/kg) improves meat quality through modulation of ruminal butyrate‐producing bacteria and activation of the IRS1/Akt/p70S6K/4EBP1 and AdipoQ signaling pathways, thereby promoting a shift of muscle fibers toward an oxidative phenotype (Shen et al. 2022). In fattening pigs, RES supplementation has likewise been reported to enhance meat color, reduce shear force, and increase IMF content (Huang et al. 2020). In Hu sheep, supplementation with Mog V (1200 mg/kg) has been shown to markedly improve growth performance, IMF content, and meat redness; in contrast, in heat‐stressed pig models, EGCG has been demonstrated to alleviate heat stress‐induced damage by suppressing oxidative stress and improving mitochondrial function, with a concomitant increase in IMF deposition (Yang et al. 2025).

Based on these findings, the present study was designed to investigate the overall physiological responses and production performance of Hu sheep under heat stress. Using dietary doses with previously validated efficacy, we systematically compared the alleviating effects of Mog V, EGCG, and RES on heat stress‐induced damage and explored their potential mechanisms in relation to changes in rumen microbiota composition and short‐chain fatty acid metabolism. To this end, forty healthy three‐month‐old male Hu sheep were randomly assigned to a control group or one of three treatment groups receiving Mog V (1200 mg/kg), EGCG (800 mg/kg), or RES (150 mg/kg). The experiment lasted 70 days, including a 10‐day adaptation period followed by a 60‐day feeding trial, with all animals managed under standardized feeding and health protocols; the composition of the basal diet is presented in Tables 1 and 2.

**TABLE 1:** Composition and nutritional levels of TMR.

TMR composition	Content/%	Nutritional levels	Content/%
Soybean meal	5.0	Dry Matter	56.62
Corn silage	48.0	Total Digestible Nutrients (TDN)	26.46
Peanut vine	11.0	Metabolizable Energy (ME, MJ/kg)^a^	6.27
Mixed bran	8.3	Crude Protein (CP)	8.79
Dried cassava distillers' grains	7.7	Crude Fat	1.75
Concentrate feed	20.0	Crude Fiber (CF)	9.23
Total	100	Neutral Detergent Fiber (NDF)	24.98
		Acid Detergent Fiber (ADF)	12.4
		Crude Ash	6.15
		Calcium (Ca)	0.30
		Total Phosphorus (P)	0.21
		Sodium Chloride (NaCl)	0.30
		Sodium (Na)	0.18
		Chloride (Cl)	0.18
		Potassium (K)	0.65
		Sulfur (S)	0.0109

^a^ The metabolizable energy in the nutritional level is the calculated value, and the rest are measured values; the same applies to the following table.

**TABLE 2:** Composition and nutritional levels of concentrate.

Concentrate composition	Content/%	Nutritional levels	Content/%
Corn	55.0	Dry Matter	87.12
Soybean meal	20.0	TDN	70.7
Wheat bran	8.0	Metabolizable Energy (ME)/(MJ/kg)^a^	12.5
Sodium bicarbonate	0.5	CP	15.3
Salt	1.2	Crude Fat/%	3.05
Mixed bran	4.7	CF	3.68
Palm kernel meal	5.0	NDF	15.84
Probiotics	0.6	ADF	6.82
Premix	5.0	Crude Ash	8.23
Total	100	Ca	0.64
		P	0.43
		NaCl	1.31
		Na	0.71
		Cl	0.77
		K	0.62
		S	0.0024

*Note:* Premix was purchased from Nanning Baichangyuan Feed Co. Ltd. (Guangxi, China).

^a^ The metabolizable energy in the nutritional level is the calculated value, and the rest are measured values; the same applies to the following table.

### 2.2 Sheep House Temperature and Humidity Measurement

A temperature and humidity sensor was positioned 1.7 m above the barn floor, and data were recorded at 30‐min intervals. Continuous curves of temperature and humidity were generated, and the THI was calculated. The degree of heat stress (HS) in the barn was evaluated according to the method of Mahjoubi et al. (2015). The THI was calculated using the following formula: $\mathrm{THI}=0.8\times T+\mathrm{RH}/100\times \left(T–14.3\right)+46.4$.

In the formula, *T* denotes ambient temperature (°C) and RH denotes relative humidity (%). Heat stress levels were classified as follows: THI ≤ 72, none; 73–77, mild; 78–89, moderate; and ≥ 90, severe.

### 2.3 Measurement of Growth Performance

The body weight of each group was recorded in the morning prior to feeding on days 0, 30, and 60 of the experiment. Average daily gain (ADG) was determined from the initial and final body weights. The amounts of feed offered and refused were recorded daily per group to compute the average daily feed intake (ADFI) and feed conversion ratio (FCR) according to the following equations:

$$\begin{array}{c} \mathrm{ADG}=\left(\text{Final body weight}–\text{Initial body weight}\right)/\\ \text{Number of experimental days} \end{array}$$

$$\begin{array}{c} \text{ADFI}=\left(\text{Total feed offered}–\text{Total refusals}\right)/\\ \text{Number of experimental days} \end{array}$$

FCR=Daily feed intake/Daily weight gain

### 2.4 Determination of RR and RT

RR and RT of each Hu sheep group were measured at 10‐day intervals. RR was assessed by visual observation, defining one inhalation–exhalation cycle as a single breath; measurements were continuously recorded for 3 min and the mean value was calculated. RT was measured using a lubricated electronic thermometer disinfected with 75% alcohol, inserted approximately two‐thirds of the rectal length, and recorded three times to calculate the average value.

### 2.5 Measurement of Blood Physiological and Biochemical Indicators

At the end of the trial, venous blood was collected from 8 Hu sheep randomly selected from each group. Five milliliters of blood were collected into anticoagulant tubes for hematological analysis. An additional 5 mL of blood was collected into non‐anticoagulant tubes, centrifuged at 3000 r/min for 15 min at 4°C to obtain serum, which was then stored at −80°C. Serum levels of HSP70, HSP90, IL‐1β, IL‐6, and TNF‐α, together with MDA, T‐AOC, SOD, CAT, and GSH‐Px activities, were measured using commercial kits (Quanzhou Jiubang Biotechnology Co. Ltd.) in accordance with the manufacturer's instructions.

### 2.6 GC–MS Analysis of SCFAs

#### 2.6.1 Collection and Pre‐Treatment of Rumen Fluid and Serum Samples

Rumen fluid and serum preparation: On the morning of the last day of the trial, six Hu sheep per group were randomly selected, and rumen fluid was collected by oral stomach tubing. The initial sample was discarded, and 50 mL of rumen fluid was collected, filtered through four layers of gauze, aliquoted into cryovials, immediately frozen in liquid nitrogen, and stored at −80°C. For pre‐treatment, 1 mL of each sample was vortexed for 30 s and centrifuged at 12,000 r/min for 10 min at 4°C. Subsequently, 100 μL of supernatant was mixed with 400 μL of ultrapure water, followed by the addition of 100 μL of 15% phosphoric acid, 20 μL of 0.375 mg/mL isocaproic acid, and 280 μL of ether. The mixture was vortexed for 1 min, centrifuged at 12,000 r/min for 10 min at 4°C, and the supernatant was collected for short‐chain fatty acids (SCFAs) analysis. For serum, 200 μL was mixed with 100 μL of 15% phosphoric acid, 20 μL of 75 μg/mL isocaproic acid, and 280 μL of ether, vortexed for 1 min, centrifuged at 4°C for 10 min, and the resulting supernatant was collected for SCFAs quantification.

#### 2.6.2 GC–MS Analysis of SCFAs

SCFAs in rumen fluid and serum were quantified using gas chromatography–mass spectrometry (GC–MS). Analyses were performed using an Agilent HP‐INNOWAX capillary column (30 m × 0.25 mm × 0.25 μm) with split injection (10:1), a 1 μL injection volume, and an injector temperature of 250°C. The oven temperature was programmed as follows: initially 90°C, ramped at 10°C/min to 120°C, then 5°C/min to 150°C, and finally 25°C/min to 250°C, which was held for 2 min. The ion source, transfer line, and quadrupole temperatures were set at 230°C, 250°C, and 150°C, respectively. Helium was employed as the carrier gas at a flow rate of 1.0 mL/min.

### 2.7 16S rRNA Sequencing

#### 2.7.1 Extraction of Microbial DNA From Rumen Fluid

DNA was extracted from rumen fluid samples using the PowerSoil DNA Isolation Kit (MOBIO Laboratories Inc., Carlsbad, CA, USA) according to the manufacturer's protocol. DNA concentration and purity were measured, and DNA integrity was evaluated by agarose gel electrophoresis.

#### 2.7.2 16S rRNA Gene Library Construction and High‐Throughput Sequencing

Total DNA extracted from rumen fluid served as the template for amplification of the full‐length bacterial 16S rRNA gene with universal primers 27F (5′‐AGRGTTTGATYNTGGCTCAG‐3′) and 1492R (5′‐TASGGHTACCTTGTTASGACTT‐3′), each appended with a unique barcode sequence. The PCR reaction was performed in a 20 μL system containing 50 ng genomic DNA, 1 μL of each primer (10 μM), 10 μL KOD One PCR Master Mix (Toyobo Co. Ltd., Osaka, Japan), 4 μL of 2.5 mM dNTPs, and nuclease‐free water to volume. The thermal cycling program was carried out as follows: initial denaturation at 95°C for 15 min, followed by 25 cycles of 98°C for 30 s, 50°C for 30 s, and 72°C for 2 min, with a final extension at 72°C for 7 min. PCR products were assessed via 1.5% agarose gel electrophoresis, purified with the PureLink kit (Thermo Fisher Scientific, Waltham, MA, USA), and quantified using a Qubit 2.0 fluorometer (Thermo Fisher Scientific, Waltham, MA, USA). Libraries that met quality control criteria were subsequently sequenced on the PacBio Sequel II platform (Pacific Biosciences of California Inc., Menlo Park, CA, USA).

#### 2.7.3 Taxonomic Analysis

Raw Circular Consensus Sequences (CCS) were demultiplexed using lima (v1.7.0) based on barcode sequences. Cutadapt (v1.9.1) was employed to remove sequences that lacked primers or fell outside the 1200–1650 bp range. Chimeric sequences were detected and eliminated using UCHIME (v4.2), resulting in high‐quality CCS reads. Sequences were clustered into operational taxonomic units (OTUs) at 97% similarity using Usearch (v10.0), and OTUs with relative abundances below 0.005% were subsequently filtered. Representative sequences were taxonomically assigned using the RDP classifier (v2.2) against the SILVA132 database with a confidence threshold of 80%, and the microbial composition and relative abundances across taxonomic levels were calculated for each sample.

#### 2.7.4 Rumen Microbiota Diversity Analysis

Venn diagrams were used to visualize shared and unique OTUs among different groups. Alpha and beta diversity analyses were conducted on 24 samples and visualized using QIIME (v1.8.0) and R (v3.2.0). Alpha diversity indices, including Chao1, ACE, Shannon, and Simpson, were calculated using mothur (v1.30.1), with Chao1 and ACE reflecting species richness, and Shannon and Simpson indices representing species diversity. Beta diversity was assessed using unweighted and weighted UniFrac distance matrices, and differences in microbial community composition among groups were assessed using principal coordinate analysis (PCoA).

#### 2.7.5 Differential Abundance Analysis

Linear discriminant analysis Effect Size (LEfSe) was used to identify taxa exhibiting statistically significant differences among treatment groups. Non‐parametric tests were conducted for intergroup comparisons at various taxonomic levels, with Linear Discriminant Analysis (LDA) scores > 3 and *p* < 0.05 considered significant.

### 2.8 Correlation Analysis

Spearman correlation analysis at the genus level was performed to assess associations between rumen microbiota and SCFA concentrations in both rumen fluid and serum.

### 2.9 Statistical Analysis

Experimental data were analyzed with SPSS 27.0. Independent sample *t*‐tests or one‐way ANOVA were initially conducted to determine statistical significance. Duncan's multiple range test was employed for post hoc comparisons. Data are presented as mean ± standard error (SE). Significance was defined as *p* < 0.05, with *p* < 0.01 considered highly significant. Graphs were created using GraphPad Prism (v9.5).

## 3 Results

### 3.1 Changes in Barn Temperature and Humidity and Effects of Three Natural Plant Extracts on Growth Performance and Physiological Parameters of Heat‐Stressed Hu Sheep

**FIGURE 1:**
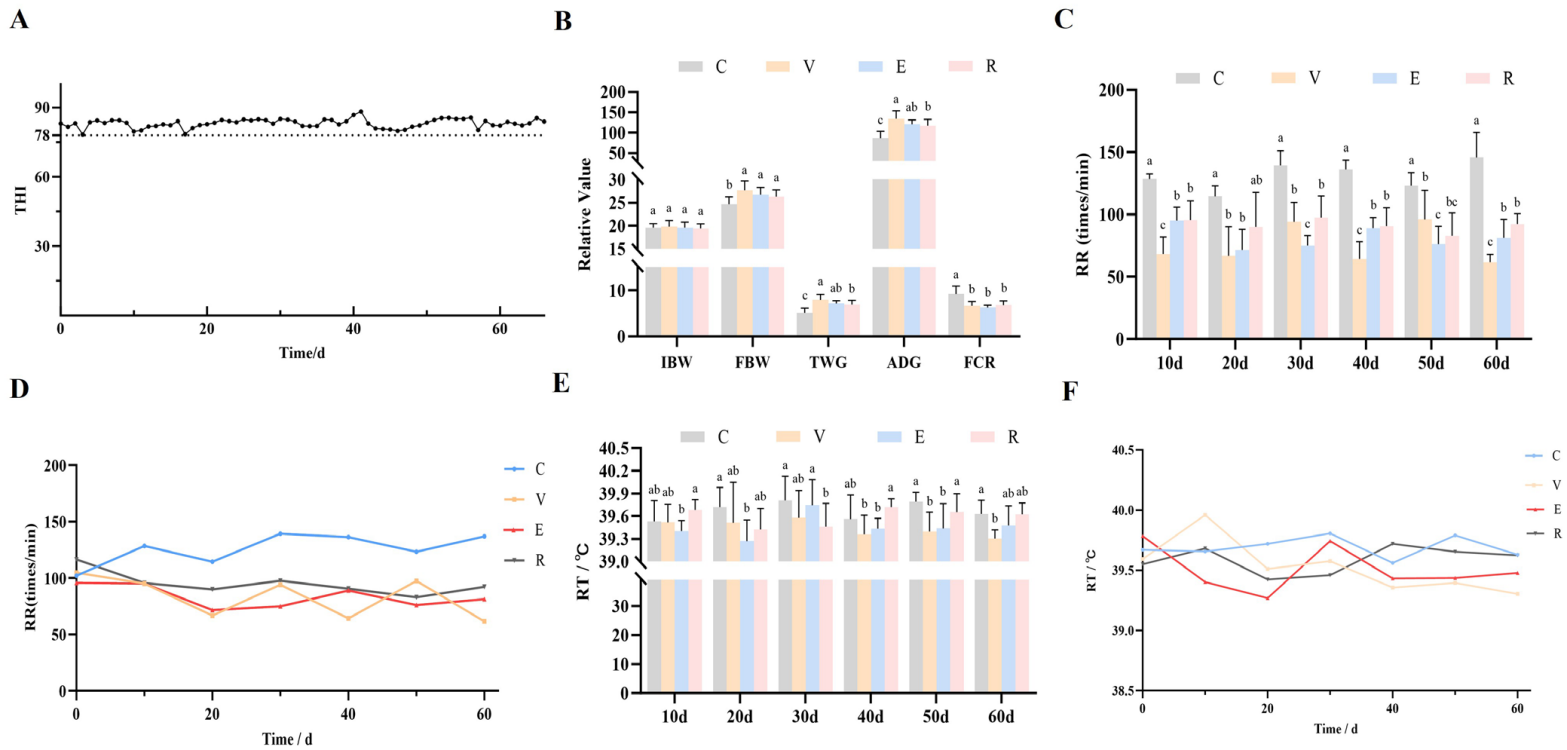
Changes in temperature and humidity in the sheep house and the effects of three natural plant extracts on the growth performance of heat‐stressed Hu sheep. (A) Changes in THI in the sheep house. (B) Effects of three natural plant extracts on the growth performance of HS Hu sheep. (C) Effects of three natural plant extracts on RR of HS Hu sheep. (D) RR line graph. (E) Effects of three natural plant extracts on RT of HS Hu sheep. (F) RT line graph. C represents the control group, V represents Mog V, E represents epigallocatechin gallate (EGCG), and R represents resveratrol (RES); different lowercase letters indicate significant differences (*p* < 0.05), and the same lowercase letters indicate insignificant differences (*p* > 0.05). The same applies below.

The barn environment was continuously monitored for temperature and humidity, and growth performance parameters—including initial body weight (IBW), final body weight (FBW), total weight gain (TWG), ADG, and feed conversion ratio (FCR)—as well as physiological indicators such as RR and RT were recorded to evaluate the effects of three natural plant extracts on the growth performance, feed efficiency, and physiological status of HS Hu sheep. As shown in Figure 1A, the THI was summarized daily from 08:00 to 18:00, and the mean THI was calculated. Moderate HS was experienced by Hu sheep throughout the trial. Figure 1B indicated that all three additives significantly influenced growth performance under HS. Initial body weights did not differ among groups; however, after supplementation, FBW in the V, E, and R groups was significantly higher than that in the C group (*p* < 0.05). Among these, the V group exhibited the highest TWG and ADG, which were higher than those in the E and R groups (*p* < 0.05). Regarding feed efficiency, FCR in the V, E, and R groups was significantly lower than that in the C group (*p* < 0.05), with the lowest FCR observed in the E group. Additionally, only the V group exhibited a significantly higher ADFI compared to the C group (*p* < 0.05), whereas no differences were observed in other treatment groups.

Figure 1D,F show the dynamic changes of RR and RT under HS. Figure 1C shows that, except for the R group on day 20 (*p* > 0.05), RR in all treatment groups was significantly lower than that in the C group at all time points (*p* < 0.05), with a 57.8% reduction in RR observed in the V group relative to C on day 60. Figure 1E,F show that RT changes were stage‐specific: no significant differences were observed among groups on day 10; on day 20, RT in the E group was significantly lower than in C (*p* < 0.05); on day 30, RT in the R group was significantly lower than in C and E (*p* < 0.05); on day 40, RT in the V and E groups was significantly lower than in R (*p* < 0.05); on day 50, RT in the V and E groups was significantly lower than in C and R (*p* < 0.05); by day 60, only the V group exhibited significant differences relative to C and R (*p* < 0.05). Collectively, the V group exhibited the most pronounced and sustained ability to regulate RR and RT throughout the trial.

### 3.2 Effects of Three Natural Plant Extracts on Blood Biochemical Parameters of HS Hu Sheep

**FIGURE 2:**
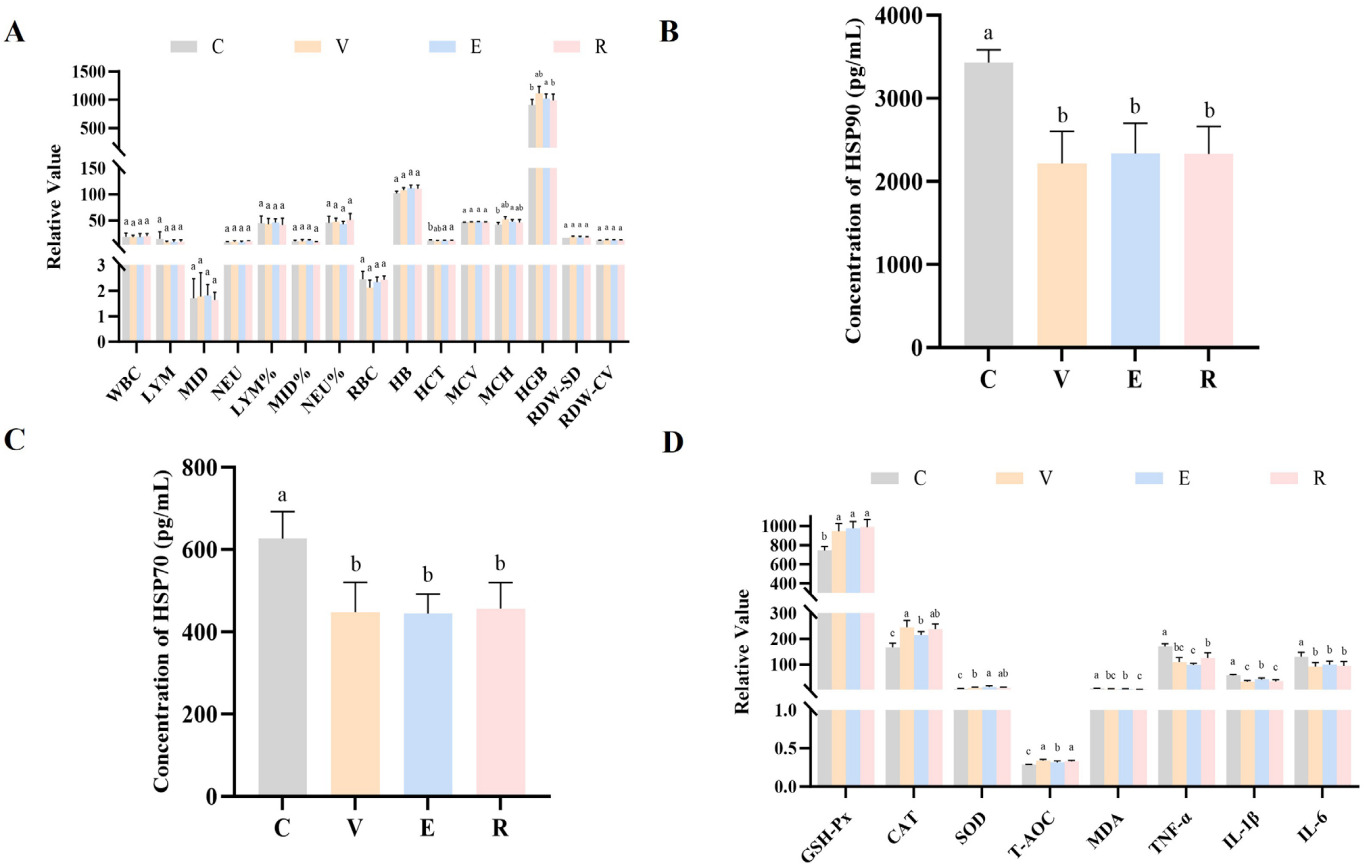
Effects of three natural plant extracts on blood parameters of heat‐stressed Hu sheep. (A) Effects of three natural plant extracts on routine blood tests of heat‐stressed Hu sheep. (B) Effects of three natural plant extracts on serum HSP70 of heat‐stressed Hu sheep. (C) Effects of three natural plant extracts on serum oxidative stress and inflammatory factor indicators of heat‐stressed Hu sheep. (D) Effects of three natural plant extracts on serum HSP90 of heat‐stressed Hu sheep.

Upon completion of the experiment, hematological analysis was performed on jugular venous blood samples from Hu sheep, and ELISA and biochemical assay kits were employed to assess the relative expression levels of serum HSP70 and HSP90, as well as serum oxidative and inflammatory indicators. According to hematological analysis (Figure 2A), several parameters were significantly different among groups under HS conditions (*p* < 0.05). For erythrocyte‐related parameters, hemoglobin concentration (HB) in the E, R, and V groups was significantly higher than in the C group (*p* < 0.05), whereas the E group exhibited a significantly greater mean corpuscular volume (MCV, *p* < 0.05). Hemoglobin content in the V group increased by 23.1% relative to the C group (*p* < 0.05). No significant differences were detected in immune‐related parameters, including total leukocyte count (WBC), lymphocyte count (LYM), and neutrophil percentage (NEU%), among groups (*p* > 0.05). Although red cell distribution width–standard deviation (RDW‐SD) and red cell distribution width–coefficient of variation (RDW‐CV) showed numerical variation, no significant differences were detected (*p* > 0.05). These results indicate that the V group exhibited the greatest increase in Hb concentration, while the E and R groups showed improvements relative to the control in Hb concentration and MCV.

As demonstrated in Figure 2B,C, the relative expression levels of HSP70 and HSP90 in serum were significantly decreased (*p* < 0.05) by all three plant extracts compared with the C group, without significant differences among the V, E, and R groups (*p* > 0.05). Figure 2D demonstrates that all three natural plant extracts significantly improved oxidative and inflammatory indicators in the serum of HS Hu sheep (*p* < 0.05). In the antioxidant system, GSH‐Px and T‐AOC levels in the V, E, and R groups were significantly higher than in the C group (*p* < 0.05), with SOD activity in the E group significantly exceeding that in the V and R groups (*p* < 0.05). CAT activity was highest in the V group (245.11 U/mL), corresponding to a 46.2% increase relative to the C group (*p* < 0.05). For oxidative damage, MDA levels were lowest in the R group, corresponding to a 57.1% reduction relative to the control (*p* < 0.05). Regarding inflammatory factors, TNF‐α and IL‐1β concentrations in the V, E, and R groups were significantly lower than in the C group (170.26 and 56.45 pg/mL, respectively; *p* < 0.05), with the greatest reductions observed in the E group.

Overall, these results suggest that the V group was most effective in enhancing CAT activity, the E group was most effective in increasing SOD activity and suppressing inflammation, while the R group was most effective at alleviating lipid peroxidation damage.

### 3.3 SCFAs Content in Rumen Fluid and Serum

**FIGURE 3:**
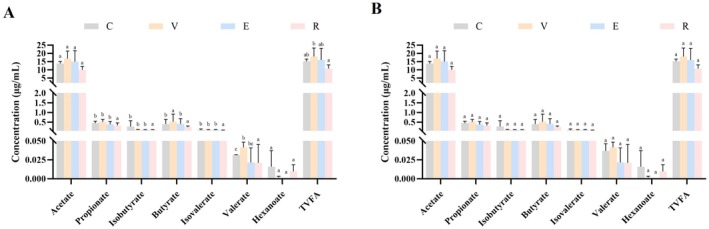
Effects of three natural plant extracts on the content of short‐chain fatty acids in rumen fluid and serum of heat‐stressed Hu sheep. (A) Comparison of SCFAs content in rumen fluid among treatment groups. (B) Comparison of short‐chain fatty acids content in serum among treatment groups.

As shown in Figure 3A, the concentrations of *propionic acid* (*propionate*), *isobutyric acid* (*isobutyrate*), *isovaleric acid* (*isovalerate*), and *valeric acid* (*valerate*) in the R group were significantly elevated compared with those in the C, V, and E groups (*p* < 0.05). Additionally, *butyrate* concentrations in the V and R groups were significantly elevated relative to the C and E groups (*p* < 0.05), with values of 658.46 ± 40.98 and 655.11 ± 17.28 μg/mL, respectively, compared with 491.11 ± 9.56 μg/mL in the C group and 514.73 ± 38.67 μg/mL in the E group. In terms of *total volatile fatty acids* (TVFA) concentration, the R group was significantly higher than the V group (*p* < 0.05) but did not differ significantly from the C and E groups (*p* > 0.05). Overall, a marked increase was observed in the production of *propionate*, *isobutyrate*, *isovalerate*, and *valerate* in the R group, whereas both the V and R groups maintained significantly higher butyrate levels than the C and E groups. These results suggest that RES may significantly enhance the concentrations of multiple SCFAs in rumen fluid by modulating rumen microbial metabolism.

As shown in Figure 3B, only minor differences in total SCFA concentrations were detected in serum among the groups; however, these differences were not statistically significant (*p* > 0.05). Likewise, no significant differences (*p* > 0.05) were observed in serum concentrations of individual SCFAs, including *acetic acid* (*acetate*), propionate, *isobutyrate*, *butyric acid* (*butyrate*), isovalerate, valerate, and *caproic acid* (*hexanoate*), among the C, V, E, and R groups. Therefore, the results indicate that the three additives did not significantly affect the serum SCFA concentrations of Hu sheep.

### 3.4 16S rRNA Gene Sequencing Results of Rumen Fluid Samples

The quality of sequencing data from rumen fluid samples was evaluated, as summarized in Table 3. A total of 1,643,459 raw CCS sequences were generated. Following quality filtering, 1,474,136 high‐quality CCS sequences were retained, with per‐sample sequence counts ranging from 228 to 440 and an average sequence length of 422 bp.

**TABLE 3:** Results of 16S rRNA gene sequencing of rumen microbiota (*n* = 6).

Group	SampleID	Raw CCS	Clean CCS	Effective‐CCS
*C*	C1	67,660	63,040	60,219
	C2	67,333	63,518	60,214
	C3	68,655	64,621	61,686
	C4	68,718	64,133	61,187
	C5	65,632	61,723	58,955
	C6	67,252	63,318	60,021
*V*	V1	59,931	56,395	54,026
	V2	72,144	67,612	64,668
	V3	77,627	72,776	70,152
	V4	75,968	71,413	68,210
	V5	65,708	61,924	59,105
	V6	73,833	68,758	64,902
*E*	E1	69,070	64,247	62,091
	E2	68,974	64,853	62,337
	E3	70,534	66,427	64,029
	E4	65,454	61,391	58,543
	E5	59,981	55,909	53,783
	E6	61,463	57,915	55,796
*R*	R1	70,763	66,096	63,281
	R2	71,392	66,476	64,242
	R3	73,387	68,723	65,819
	R4	58,123	54,653	52,340
	R5	70,056	65,846	63,057
	R6	73,801	68,312	65,473

*Note:* SampleID is the sample name; Raw‐CCS is the number of CCSs identified for the sample; CleanCCS is the number of sequences after identification and primer removal; Effective‐CCS is the number of sequences used for subsequent analysis after length filtering and removal of chimeras.

#### 3.4.1 Venn Diagram, Rarefaction Curve, Alpha Diversity, and β Diversity Analysis

**FIGURE 4:**
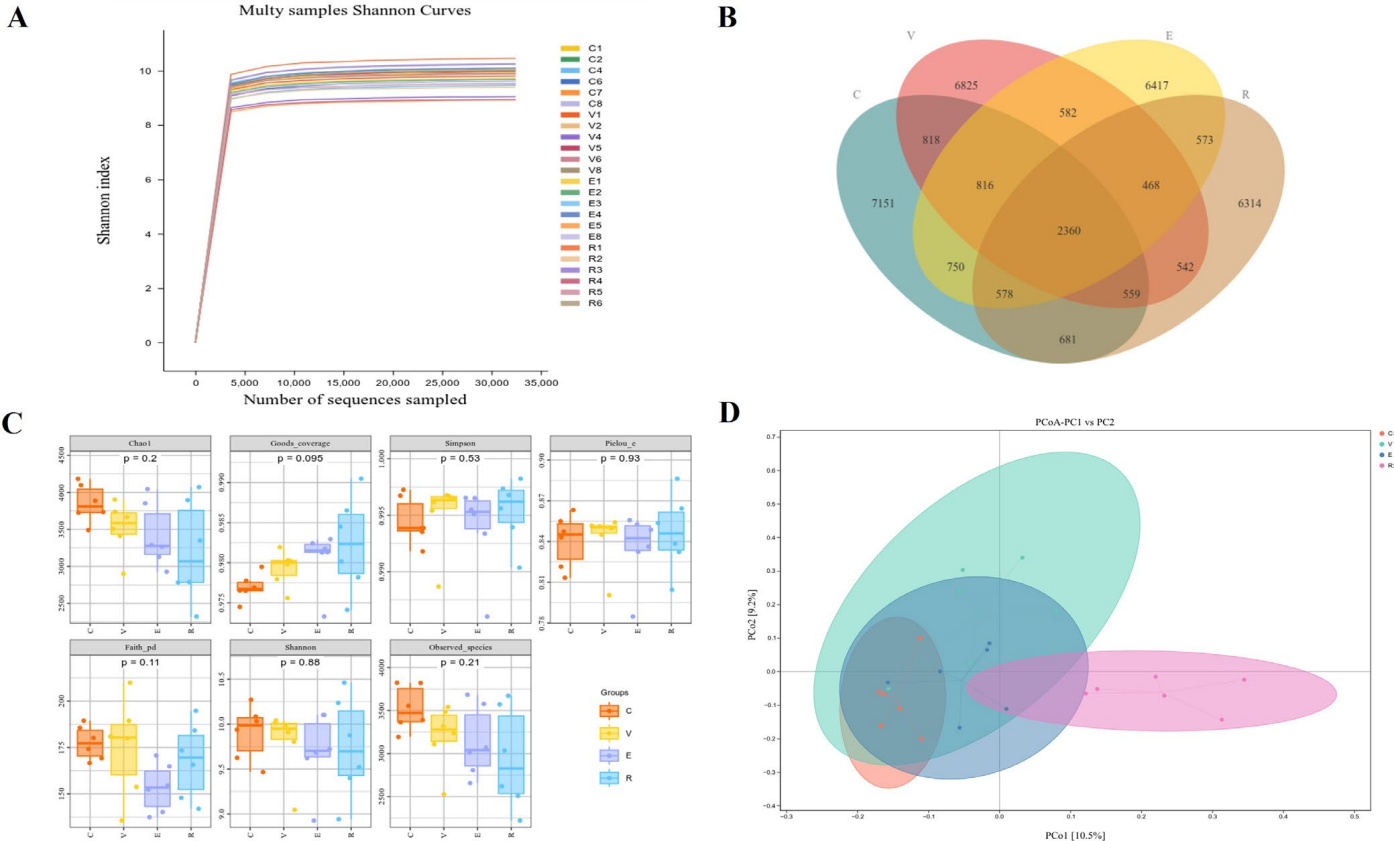
Effects of different natural additives on rumen microbial diversity and community structure of heat‐stressed Hu sheep. (A) Shannon index‐based rarefaction curve. (B) Venn diagram of OTU distribution. (C) Effects of different additives on rumen microbial α‐diversity indices in Hu sheep. (D) Principal coordinate analysis (PCoA) of rumen microbial communities based on Bray–Curtis distance.

The Venn diagram and rarefaction curve analyses are shown in Figure 4. As the number of sequencing reads increased, the rarefaction curves of the rumen microbiota in the four groups (Figure 4A) rose steeply before stabilizing, indicating that the sequencing depth adequately captured the predominant microbial taxa and reliably reflected the species richness in rumen fluid. The Venn diagram results (Figure 4B) showed that 6374, 6367, 5506, and 5259 unique OTUs were detected in the C, V, E, and R groups, respectively, indicating notable differences in the rumen microbial community structure among the treatment groups. Alpha diversity indices of the rumen microbiota (Observed_species, Chao1, Shannon, and Simpson) are shown in Figure 4C (detailed statistical results are provided in the Table S1). Compared with the C group, the Chao1 and Observed_species indices in the E and R groups were significantly lower (*p* < 0.05); compared with the V group, the R group was also significantly lower in these two indices (*p* < 0.05). No significant differences (*p* > 0.05) were detected among the four groups for the Shannon and Simpson indices. PCoA based on the Bray–Curtis distance matrix at the OTU level (Figure 4D) revealed distinct separation of the rumen microbial communities between the C and R groups, while the V and E groups overlapped, suggesting that RES intervention markedly influenced the rumen microbial community structure in Hu sheep.

#### 3.4.2 Analysis of Rumen Bacterial Composition at the Phylum Level

The composition of rumen bacterial communities at the phylum level is shown in Table 4. The results indicated that Bacteroidetes and Firmicutes were the dominant phyla, collectively accounting for 90.64%–94.53% of the relative abundance. Among these, the abundance of Bacteroidetes in the E and R groups was significantly higher than in the V group, but did not differ significantly from that in the C group (*p* > 0.05). Firmicutes abundance was highest in the V group, significantly exceeding that in the R group (*p* < 0.05), but not differing significantly from that in the C group (*p* > 0.05). Among the subdominant phyla, *Verrucomicrobia* abundance in the R group was significantly higher than in all other groups (*p* < 0.05); TM7 abundance was significantly higher than in the V and E groups (*p* < 0.05); Fibrobacteres abundance was significantly higher than in the V group (*p* < 0.05), but did not differ significantly from that in the C and E groups (*p* > 0.05). No significant differences were observed in the relative abundances of other phyla (*Tenericutes, Proteobacteria, Actinobacteria, Spirochaetes*, and *SR1*) among groups (*p* > 0.05). Additionally, the “Others” category in the R group was significantly higher than in the E group (*p* < 0.05), but did not differ significantly from the C and V groups (*p* > 0.05). In summary, the V group may have significantly altered rumen microbial structure by suppressing Bacteroidetes and increasing Firmicutes relative abundance; the R group markedly enriched *Verrucomicrobia* and *Fibrobacteres*, both known to be linked to fiber degradation, suggesting a specific regulatory effect; the E group exhibited a similar trend to the R group in Bacteroidetes modulation, albeit with weaker intensity and without statistical significance.

**TABLE 4:** Bacterial community composition of rumen microbiota at the phylum level (*n* = 6).

Item (%)	*C*	*V*	*E*	*R*
Bacteroidetes	68.61 ± 1.12^ab^	62.77 ± 3.71^b^	71.36 ± 2.18^a^	72.17 ± 0.66^a^
Firmicutes	24.03 ± 1.92^ab^	28.16 ± 3.74^a^	21.6 ± 1.44^ab^	18.03 ± 0.48^b^
Tenericutes	1.91 ± 0.42	1.62 ± 0.2	2.45 ± 1.18	3.08 ± 0.44
Proteobacteria	1.72 ± 0.42	1.71 ± 0.78	1.5 ± 0.27	1.17 ± 0.14
Verrucomicrobia	0.79 ± 0.08^b^	0.81 ± 0.22^b^	0.78 ± 0.13^b^	1.45 ± 0.21^a^
Actinobacteria	0.53 ± 0.37	2.38 ± 1.67	0.11 ± 0.03	0.11 ± 0.02
Spirochaetes	0.7 ± 0.13	0.67 ± 0.15	0.6 ± 0.12	1.02 ± 0.18
TM7	0.37 ± 0.05^ab^	0.66 ± 0.13^b^	0.38 ± 0.04^b^	0.86 ± 0.19^a^
Fibrobacteres	0.35 ± 0.07^ab^	0.27 ± 0.06^b^	0.44 ± 0.09^ab^	0.8 ± 0.29^a^
SR1	0.26 ± 0.08	0.26 ± 0.04	0.24 ± 0.06	0.41 ± 0.07
Others	0.73 ± 0.06^ab^	0.69 ± 0.12^ab^	0.53 ± 0.06^b^	0.9 ± 0.13^a^

*Note:* Data in the same industry with different lowercase letters indicate significant differences (*p* < 0.05), while data with the same lowercase letters indicate no significant differences (*p* > 0.05).

#### 3.4.3 Analysis of Rumen Bacterial Composition at the Genus Level

Analysis of the rumen bacterial composition at the genus level (Table 5) revealed significant differences in the microbial community structures among the C, V, E, and R groups (*p* < 0.05). The dominant genus *Prevotella* was significantly more abundant in the C and E groups than in the V and R groups, whereas *Succiniclasticum* abundance was highest in the V group, being significantly higher than that in all other groups. The R group exhibited significantly higher abundances of *Lachnospira*, *Anaeroplasma*, and *Oscillospira* compared with the other treatment groups. Notably, the abundance of the “Others” category in the R group was significantly higher than in all other groups.

**TABLE 5:** Bacterial community composition of rumen microbiota at the genus level (*n* = 6).

Item (%)	*C*	*V*	*E*	*R*
*Prevotella*	49.94 ± 2.28^a^	40.79 ± 3.59^b^	53.09 ± 1.53^a^	37.64 ± 2^b^
*Succiniclasticum*	4.39 ± 0.66^b^	7.07 ± 1.41^a^	3.82 ± 0.54^b^	2.83 ± 0.54^b^
*Ruminococcus*	2.99 ± 0.48^ab^	4.09 ± 0.94^a^	3.7 ± 0.91^a^	1.35 ± 0.19^b^
*Anaerostipes*	3.11 ± 1.11^a^	0.94 ± 0.25^ab^	2.27 ± 1.24^ab^	0.07 ± 0.02^b^
*CF231*	0.88 ± 0.11^b^	1.07 ± 0.16^b^	1.15 ± 0.19^b^	2.98 ± 0.38^a^
*Coprococcus*	0.8 ± 0.29^b^	1.05 ± 0.13^ab^	1.88 ± 0.59^a^	0.42 ± 0.1^b^
*RFN20*	1.29 ± 0.27^a^	0.64 ± 0.17^b^	0.61 ± 0.09^b^	0.94 ± 0.22^ab^
*Clostridiaceae_Clostridium*	0.62 ± 0.05^b^	0.67 ± 0.05^b^	0.61 ± 0.07^b^	0.91 ± 0.07^a^
*Anaeroplasma*	0.39 ± 0.08^b^	0.39 ± 0.14^b^	0.28 ± 0.04^b^	1.53 ± 0.28^a^
*Butyrivibrio*	0.72 ± 0.22^ab^	0.97 ± 0.28^a^	0.25 ± 0.03^b^	0.37 ± 0.05^b^
*Oscillospira*	0.28 ± 0.04^b^	0.4 ± 0.12^b^	0.4 ± 0.09^b^	0.99 ± 0.21^a^
*Ruminobacter*	0.9 ± 0.27^a^	0.19 ± 0.08^b^	0.65 ± 0.2^ab^	0.18 ± 0.05^b^
*Lachnospira*	0.41 ± 0.13^b^	0.19 ± 0.05^b^	0.22 ± 0.07^b^	1.09 ± 0.37^a^
*Fibrobacter*	0.34 ± 0.08^ab^	0.27 ± 0.06^b^	0.44 ± 0.09^ab^	0.8 ± 0.29^a^
*Selenomonas*	0.1 ± 0.05^ab^	0.28 ± 0.16^a^	0.09 ± 0.06^ab^	0 ± 0^b^
*Anaerovibrio*	0.06 ± 0.01^b^	0.26 ± 0.07^a^	0.03 ± 0.01^b^	0.07 ± 0.03^b^
*Desulfovibrio*	0.12 ± 0.02^ab^	0.17 ± 0.02^a^	0.08 ± 0.01^bc^	0.06 ± 0.01^b^
*Acetobacter*	0.06 ± 0.01^ab^	0.02 ± 0^b^	0.1 ± 0.02^a^	0.1 ± 0.02^a^
*Lactobacillus*	0.05 ± 0.01^ab^	0.03 ± 0.01^b^	0.09 ± 0.02^a^	0.1 ± 0.02^a^
*Lachnospiraceae_Clostridium*	0.03 ± 0.01^ab^	0.04 ± 0.01^ab^	0.02 ± 0.01^b^	0.06 ± 0.02^a^
*Others*	27.73 ± 1.55^bc^	33.78 ± 2.64^b^	25.63 ± 1.33^c^	42.59 ± 2.76^a^

*Note:* Data in the same industry with different lowercase letters indicate significant differences (*p* < 0.05), while data with the same lowercase letters indicate no significant differences (*p * > 0.05).

#### 3.4.4 LEfSe Analysis

**FIGURE 5:**
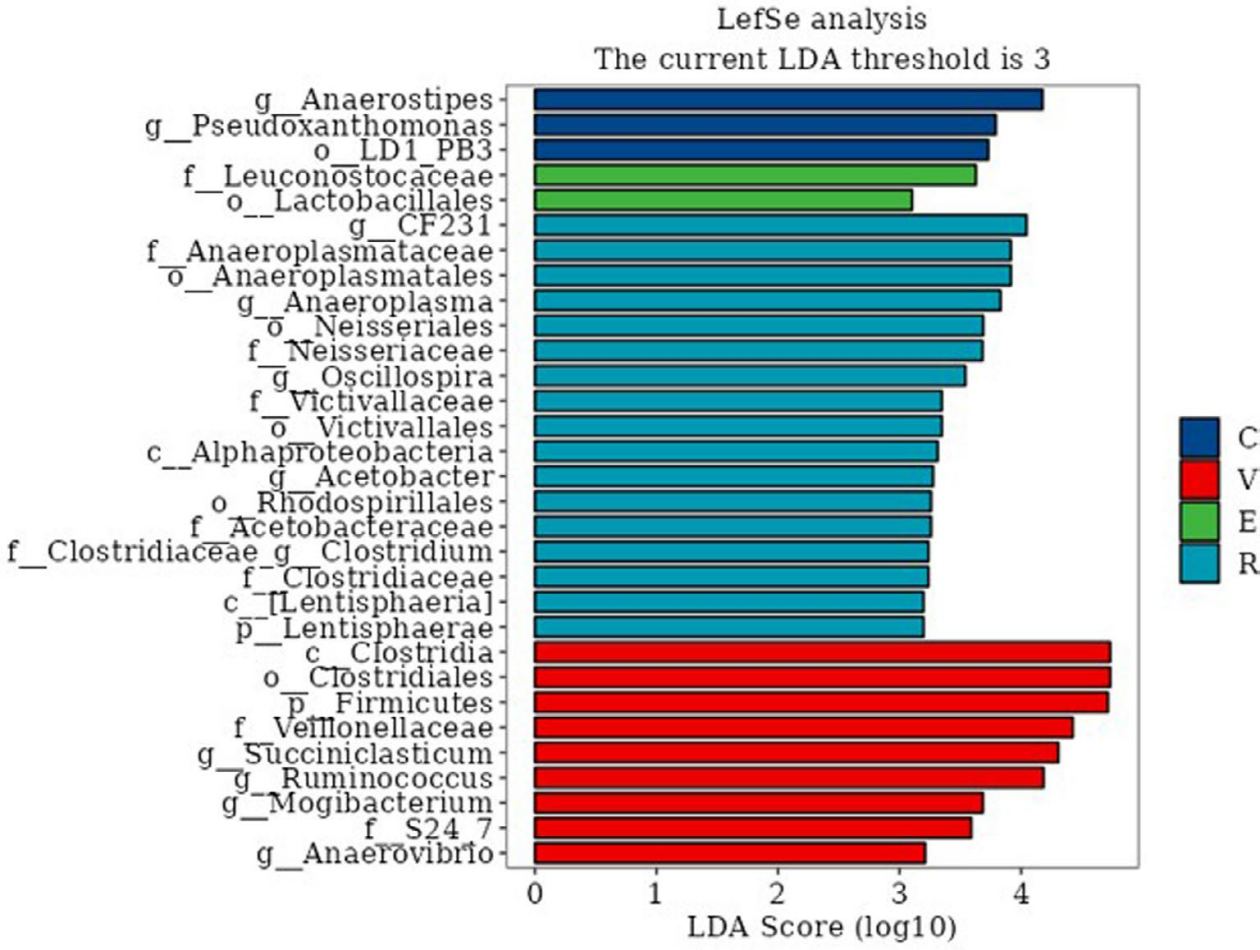
LEfSe analysis of discriminative taxa in rumen microbiota.

In this study, LEfSe analysis (LDA = 3) identified 12 bacterial genera as characteristic taxa differentiating the treatment groups (Figure 5). Specifically, the C group had two characteristic genera, the V group had six, and the R group had five. The C group was characterized by *g_Anaerostipes* and *g_Pseudoxanthomonas*. The E group was enriched in *f_Leuconostocaceae* and *o_Lactobacillales*. The R group showed enrichment of g_CF231, *f_Anaeroplasmataceae*, *o_Anaeroplasmatales*, *g_Anaeroplasma*, *o_Neisseriales*, *f_Neisseriaceae*, *g_Oscillospira*, *f_Victivallaceae*, *o_Victivallales*, *AlphaProteobacteria*, *g_Acetobacter*, *o_Rhodospirillales*, *f_Acetobacteraceae*, *f_Clostridiaceae_g_Clostridium*, *f_Clostridiaceae*, *c_Lentisphaeria*, and *p_Lentisphaerae*. The V group exhibited enrichment in *c_Clostridia*, *o_Clostridiales*, *p_Firmicutes*, *f_Veillonellaceae*, *g_Succiniclasticum*, *g_Ruminococcus*, *g_Mogibacterium*, *f_S24‐7*, and *g_Anaerovibrio*.

### 3.5 Correlation Analysis Between Rumen Microbiota and SCFA Contents in Hu Sheep

Spearman's correlation analysis was performed at the genus level to evaluate the relationships between rumen microbiota and SCFA concentrations in rumen fluid and serum of Hu sheep among different treatment groups. The results indicated distinct association patterns between the relative abundance of bacterial genera and SCFA concentrations. The heatmap (*x*‐axis) illustrated SCFA components, including *acetate*, *propionate*, *butyrate*, *isobutyrate*, *isovalerate*, *valerate*, *caproate*, and TVFA.

#### 3.5.1 Correlation Analysis of Group V

**FIGURE 6:**
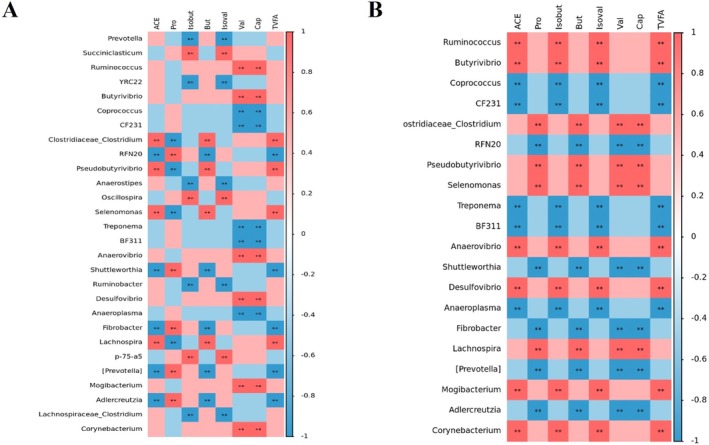
Spearman correlation analysis between rumen bacterial genera and SCFA concentrations in the V Group. (A) Spearman correlation between rumen bacterial genera and SCFA concentrations in rumen fluid of the V group. (B) Spearman correlation between rumen bacterial genera and SCFA concentrations in serum samples of the V group. The *y*‐axis represents rumen bacterial genera. The color gradient ranges from deep blue (*ρ* = −1, strongest negative correlation) to deep red (*ρ* = 1, strongest positive correlation). Statistical significance is indicated by asterisks (*p* < 0.05, **p < 0.01), similarly hereafter.

The Spearman's correlation results for the V group (Figure 6A,B) revealed significant associations between rumen bacterial genera and SCFA concentrations in both rumen fluid and serum. In rumen fluid, acetate, butyrate, and TVFA were positively correlated with several genera, such as *Clostridiaceae_Clostridium*, *PseudoButyrivibrio*, *Selenomonas*, and *Lachnospira*, and negatively correlated with RFN20, *Shuttleworthia*, and *Fibrobacter*. Propionate was negatively correlated with these genera. Moreover, *isobutyrate* and isovalerate were positively correlated with *Succiniclasticum* and *Oscillospira*.

In serum, the correlation patterns differed from those observed in rumen fluid. For instance, *Ruminococcus* and *Butyrivibrio* were positively correlated with acetate, *isobutyrate*, *isovalerate*, and TVFA, whereas *Clostridiaceae_Clostridium* and *PseudoButyrivibrio* were positively correlated with propionate and butyrate. These findings offer detailed insights into the interactions between specific bacterial taxa and key metabolic products under Mog V supplementation.

#### 3.5.2 Correlation Analysis of Group E

**FIGURE 7:**
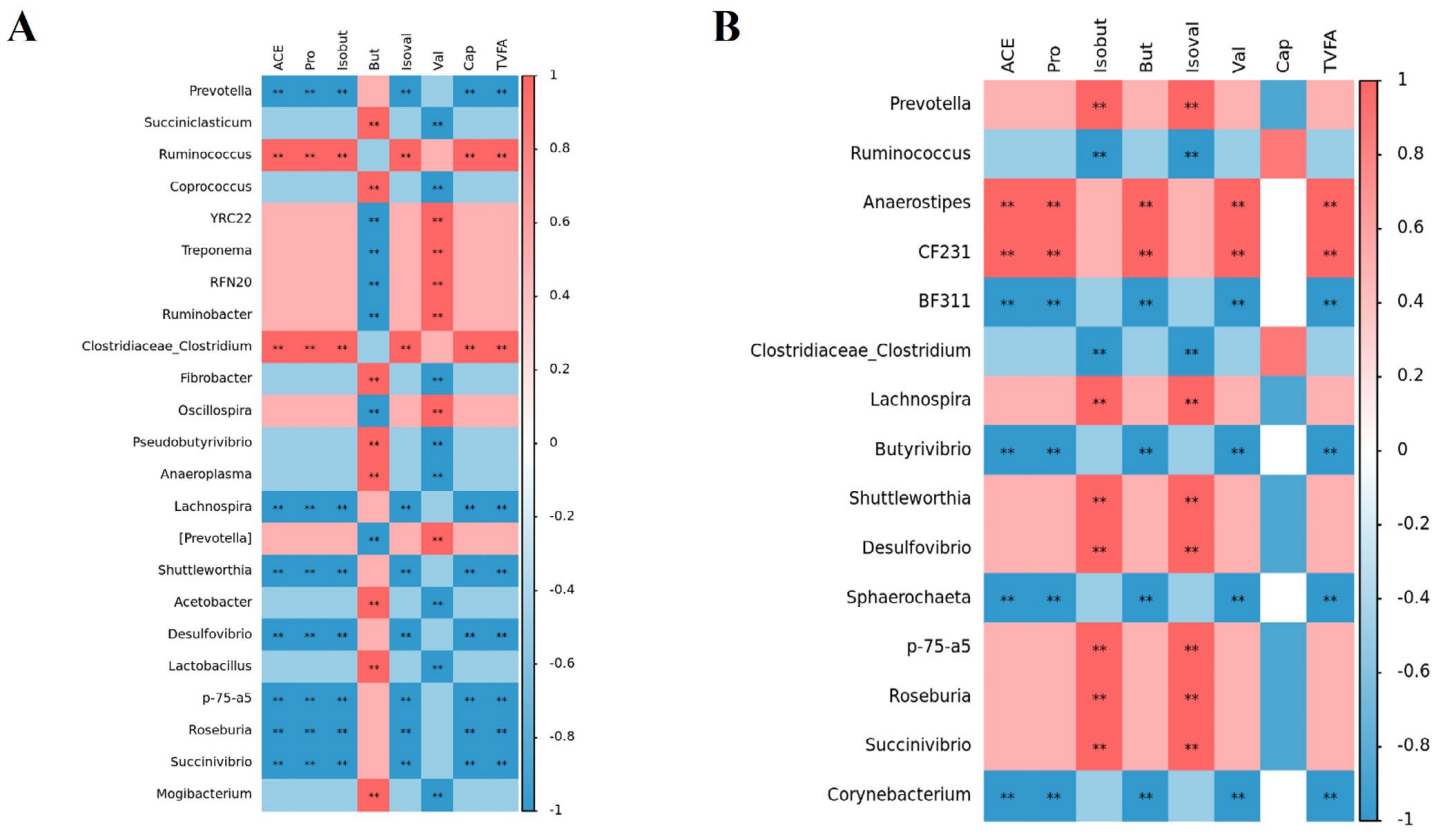
Spearman correlation analysis between rumen bacterial genera and SCFA concentrations in the E group. (A) Spearman correlation between rumen bacterial genera and SCFA concentrations in rumen fluid of the E group. (B) Spearman correlation between rumen bacterial genera and SCFA concentrations in serum samples of the E group.

The Spearman correlation analysis (Figure 7A,B) was performed to determine the associations between the relative abundance of rumen bacterial genera and the concentrations of SCFAs in rumen fluid and serum samples from the EGCG‐treated group. In rumen fluid, acetate, propionate, butyrate, valerate, caproate, and TVFA were significantly positively correlated with *Ruminococcus* and *Clostridiaceae_Clostridium*, and significantly negatively correlated with *Prevotella*, *Lachnospira*, *Shuttleworthia*, and Desulfovibrio. Butyrate showed significant positive correlations with *Succiniclasticum*, *Coprococcus*, *Fibrobacter*, *Pseudobutyrivibrio*, and Lactobacillus, whereas *valerate* showed mainly positive correlations with *YRC22*, *Anaerostipes*, *CF231*, Treponema, and *Oscillospira*. Notably, the bacterial genera associated with butyrate and valerate exhibited largely opposite correlation patterns. In serum, acetate, propionate, butyrate, valerate, and TVFA were significantly positively correlated with *Anaerostipes* and *CF231*, and significantly negatively correlated with *Butyrivibrio* and *Sphaerochaeta*. In contrast, i*sobutyrate* and *isovalerate* showed significant positive correlations with *Prevotella*, *Lachnospira*, and *Shuttleworthia*, and significantly negative correlations with *Ruminococcus* and *Clostridiaceae_Clostridium*.

#### 3.5.3 Correlation Analysis of Group R

**FIGURE 8:**
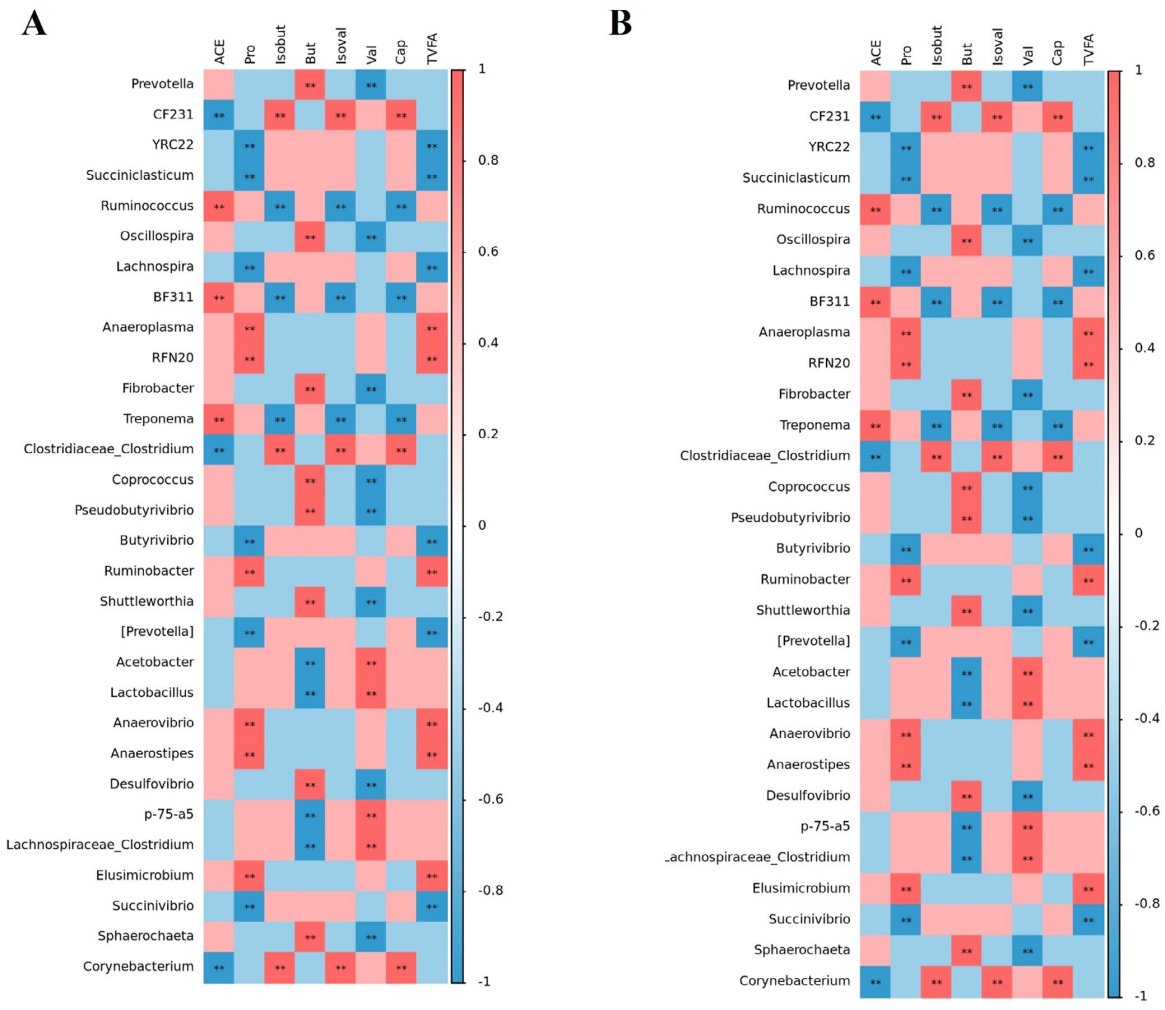
Spearman correlation analysis between rumen bacterial genera and SCFA concentrations in the R group. (A) Spearman correlation between rumen bacterial genera and SCFA concentrations in rumen fluid of the R group. (B) Spearman correlation between rumen bacterial genera and SCFA concentrations in serum samples of the R group.

Spearman correlation analysis (Figure 8A,B) revealed distinct patterns of association between the relative abundance of rumen bacterial genera and SCFA concentrations in rumen fluid and serum of the R group. In rumen fluid, acetate showed significant positive correlations with *Ruminococcus*, *BF311*, and Treponema, whereas *Isobutyrate*, *Isovalerat*, and caproate were primarily correlated with *CF231*, *Clostridiaceae_Clostridium*, and Corynebacterium. These two groups of SCFAs exhibited broadly opposite bacterial association patterns. Propionate and TVFA showed significant correlations with *Anaeroplasma*, *RFN20*, *Ruminobacter*, *Anaerovibrio*, and *Anaerostipes*. Butyrate was predominantly associated with *Prevotella*, *Oscillospira*, *Fibrobacter*, *Coprococcus*, and *PseudoButyrivibrio*, while the bacterial genera associated with valerate (e.g., Acetobacter, Lactobacillus, p‐75‐a5) exhibited an overall inverse association pattern relative to those associated with butyrate. In serum, acetate, propionate, *Isobutyrate*, Valerate, and TVFA showed significant correlations with genera such as *YRC22*, *Succiniclasticum*, *Lachnospira*, *Butyrivibrio*, and [*Prevotella*], corresponding to the genera that were negatively associated with propionate and TVFA in rumen fluid. The correlation patterns of *isovalerat*, *caproate*, and *butyrate* in serum largely mirrored the corresponding patterns observed in rumen fluid.

## 4 Discussion

HS has been shown to disrupt thermoregulatory homeostasis and markedly impair growth and immune function. In this study, a THI > 78 combined with elevated RR (> 120 breaths/min), increased RT (> 39.5°C), and elevated serum HSP70 levels (> 600 pg/mL) collectively confirmed a severe heat‐stress state. Although the age and sex of the animals used in the study by Zou et al. (2025) differed, precluding direct comparison of absolute values, the consistent physiological trends (RR ≈ 29.6; RT ≈ 39.19°C under thermoneutral conditions) support the validity of the heat‐stress model adopted here.

Dietary supplementation with Mog V, EGCG, or RES significantly enhanced growth performance, including TWG, ADG, feed intake, and FCR, consistent with previous studies (Liu, Lu, et al. 2025; Liu, Ge, et al. 2025; Shen et al. 2022; Thorne et al. 2021). These improvements may be attributable to enhanced antioxidant capacity, improved nutrient utilization, and strengthened immune function. All three extracts significantly reduced respiratory rate (*p* < 0.05), with Mog V exerting the most pronounced effect, consistent with its reported antitussive activity (Wu et al. 2022). EGCG and RES likely exert their effects through anti‐inflammatory and antioxidant pathways (He et al. 2019; Jiang et al. 2019; Penalva et al. 2015).

Hematological parameters related to hemoglobin were significantly elevated without inducing adverse effects (Ding et al. 2024; Zhang, Ma, et al. 2022; Zhang, Zheng, et al. 2022). The significant reductions in HSP70/HSP90 levels (*p* < 0.01) indicated an alleviation of cellular stress. Consistent with previous reports describing the ability of Mog V, EGCG, and RES to enhance antioxidant enzyme activity via the Nrf2/HO‐1, TLR4/MAPK/NF‐κB, or SIRT1‐related pathways (Du et al. 2022; Luo et al. 2018; Mi et al. 2018; Mo et al. 2021; Rodríguez‐Enríquez et al. 2019), our results demonstrated increased T‐AOC, T‐SOD, and GSH‐Px activities and a concomitant decrease in MDA content.

Similarly, all three extracts significantly decreased serum IL‐1β, IL‐6, and TNF‐α concentrations (*p* < 0.01), consistent with reported mechanisms involving the COX‐2/IL‐6, NF‐κB/STAT3, and TLR4/JAK/STAT pathways (Lee et al. 2023; Mokra et al. 2022; Shi et al. 2025; Xu et al. 2024). Collectively, Mog V, EGCG, and RES effectively mitigated heat‐stress‐induced physiological disturbances, oxidative damage, and inflammatory responses, highlighting their potential as functional additives for heat‐stress management in ruminants.

To further elucidate the underlying mechanisms, this study systematically assessed the regulatory effects of the three extracts on rumen microbiota and SCFA metabolism using α‐ and β‐diversity analyses, LEfSe, and Spearman correlation. The α‐diversity analysis revealed significant reductions in the Chao1 and Observed species indices in the E and R groups (*p* < 0.05), consistent with the potential antibacterial properties of polyphenols (Che et al. 2024; Shi et al. 2025), whereas the Shannon and Simpson indices remained unchanged (*p* > 0.05), suggesting that community evenness may be maintained by core microbial redundancy (Jia et al. 2024). The β‐diversity analysis further demonstrated that RES markedly reshaped the microbial community structure, highlighting its potential to modulate the rumen microecology.

LEfSe analysis identified 12 differential genera that clearly distinguished microbial communities among the treatments. In the V group, Firmicutes and butyrate‐producing genera (e.g., *Succiniclasticum* and *Ruminococcus*) were significantly enriched, suggesting enhanced fiber degradation and butyrate synthesis capacity. Similarly, Li et al. (2025) reported that the rumen microbiota of heat‐tolerant dairy cows was dominated by *Ruminococcus flavefaciens* and *Succiniclasticum*, which play essential roles in fiber degradation and SCFA production and are important for thermotolerance regulation. The increase in Firmicutes was also consistent with the findings of Wang et al. (2022). These findings indicate effective alleviation of HS and suggest that Mog V may mitigate HS‐induced growth performance decline by modulating both blood biochemical markers and rumen microbial composition in Hu sheep. Moreover, butyrate, as a major energy source for colonic epithelial cells, can suppress inflammatory cytokine release through activation of the GPR43 receptor (Pirozzi et al. 2018), which is consistent with the decreased serum IL‐6 concentrations observed in the V group. The reduced F:G ratio further indicated improved energy utilization efficiency.

EGCG treatment resulted in the enrichment of *Lactobacillales* and *Leuconostocaceae*. *Lactobacillales* are known to maintain microbial balance mainly through lactic acid production and pathogen inhibition in the rumen and intestinal ecosystem, while also contributing to energy metabolism and host health regulation (Makarova et al. 2006). *Leuconostocaceae* are characterized by lactic acid and ethanol production and are often associated with the fermentation of complex carbohydrates and flavor compound formation (Zheng et al. 2020). Their involvement in lactic acid metabolism may lower pH, inhibit opportunistic pathogens such as *Prevotella* (Pu et al. 2023; Zeng et al. 2023), and be accompanied by increased acetate concentrations.

In the R group, enrichment of *Verrucomicrobia* and *Fibrobacteres* was observed. *Verrucomicrobia* play important roles in maintaining intestinal barrier function and immune homeostasis, while *Fibrobacteres* are core cellulolytic taxa in the rumen and cecum of herbivores, essential for cellulose degradation and volatile fatty acid production (de Vos 2017; Ransom‐Jones et al. 2012). Furthermore, a genus within *Verrucomicrobia*—*Oscillospira*—was significantly positively correlated with butyrate. Butyrate can activate the Nrf2/ARE pathway and enhance the activities of antioxidant enzymes such as SOD and GSH‐Px (Guo et al. 2020), suggesting that RES may synergistically enhance antioxidant capacity and energy metabolism.

Correlation analysis further revealed the influence of the three extracts on SCFA–related metabolic pathways. In the V group, butyrate showed positive correlations with *Clostridiaceae_Clostridium* and *PseudoButyrivibrio*. Members of *Clostridiaceae* (particularly Clostridium spp.) are recognized butyrate producers and symbionts that play essential roles in preserving the intestinal barrier, regulating immune function, and mitigating inflammation (Guo et al. 2020). Certain strains, such as *C. butyricum* , are employed as probiotics to enhance intestinal health and reduce the risk of *Clostridium difficile* infection. *PseudoButyrivibrio* species are predominantly detected in the ruminant gastrointestinal tract, where they contribute to cellulose and hemicellulose degradation, butyrate formation, protein breakdown, and lipid biohydrogenation. These functions collectively enhance digestive efficiency and nutrient absorption. These findings suggest that the enriched taxa in this group may increase cellulase activity to promote butyrate production, while simultaneously suppressing propionate‐producing *Prevotella*, thereby decreasing the risk of ruminal acidosis (Zhang, Ma, et al. 2022; Zhang, Zheng, et al. 2022).

In the E group, acetate exhibited positive correlations with *Ruminococcus* and *Clostridiaceae_Clostridium*, but negative correlations with *Prevotella* and *Lachnospira*. *Ruminococcus* can degrade complex polysaccharides and produce SCFAs (e.g., butyrate), thereby contributing to intestinal energy metabolism and barrier function. *Clostridiaceae_Clostridium* are closely involved in protein and amino acid fermentation and can produce butyrate and other metabolites, although certain strains may also be linked to inflammation or toxin production (Flint et al. 2012). Conversely, reduced abundances of Prevotella and *Lachnospira* are associated with lower chronic intestinal inflammation and improved metabolic health, potentially reducing the risks of obesity, dysglycemia, and cancer (Liu, Lu, et al. 2025; Liu, Ge, et al. 2025; Okalin et al. 2025). These results indicate that the antimicrobial activity of EGCG may help maintain a stable ruminal environment (Guo et al. 2023; Zhu et al. 2024). Moreover, EGCG may attenuate inflammation through GPR43 activation, consistent with the observed reduction in serum inflammatory cytokines (Che et al. 2024).

In the R group, acetate was positively correlated with *Ruminococcus*, whereas isovalerate and hexanoate (valerate) were strongly associated with *Clostridiaceae* and Corynebacterium. *Ruminococcus* species in the human gut are reported to degrade complex dietary fibers (e.g., resistant starch) (La Reau and Suen 2018), thereby producing SCFAs (particularly butyrate) that promote intestinal health (Tran and Mohajeri 2021). The associations of isovalerate and hexanoate with Clostridiaceae and Corynebacterium suggest that these microbial metabolites may participate in modulating the gut environment, maintaining metabolic homeostasis, and exerting anti‐inflammatory and gut‐protective effects via inhibition of the histone deacetylase (HDAC) pathway (Guo et al. 2020; Tran and Mohajeri 2021; Yuille et al. 2018). These findings indicate that RES remodels the energy metabolism network by regulating key SCFA‐producing genera. Previous studies have reported that RES supplementation increases beneficial ruminal or intestinal genera (e.g., *Acetitomaculum*, *Moryella*, *Lactobacillus*, *Akkermansia*), thereby improving antioxidant status and growth performance (Chen et al. 2016; Li et al. 2024; Shen et al. 2022).

Taken together, the three natural extracts were found to mitigate HS in Hu sheep via distinct mechanisms, primarily through modulation of the “microbiota–metabolite–host” interaction network. Mog V enriched Firmicutes and butyrate‐producing genera (e.g., *Ruminococcus, Succiniclasticum*), thereby promoting fiber degradation and butyrate production, which were associated with suppression of inflammation via GPR43 activation and enhancement of antioxidant capacity through the Nrf2/ARE pathway. EGCG was associated with enrichment of *Lactobacillales* and *Streptococcaceae*, increased acetate production, and reduced ruminal pH, thereby inhibiting opportunistic pathogens (e.g., *Prevotella*), while acetate‐mediated GPR43 activation likely contributed to microbial stability and immune optimization. RES increased the abundance of *Verrucomicrobia* and *Fibrobacteres*, facilitating fiber degradation and the production of butyrate and branched‐chain fatty acids (e.g., *isovalerate*), which were linked to enhanced antioxidant defenses and epithelial integrity, potentially via HDAC inhibition. Collectively, Mog V predominantly exerted butyrate‐mediated protective effects, EGCG was associated with acetate‐linked microbial homeostasis, whereas RES facilitated the integration of fiber degradation with multi–fatty acid metabolism, resulting in broader antioxidant and barrier‐protective outcomes.

Several limitations of the present study should be acknowledged. First, antioxidant‐treated groups under non–heat‐stress conditions were not included, and meat quality traits were not directly assessed in Hu sheep; thus, the differential effects of plant‐derived antioxidants on meat quality across environmental conditions could not be systematically evaluated. Nevertheless, our previous studies have comprehensively assessed the effects of Mog V, EGCG, and RES on meat quality and intramuscular fat deposition in multiple animal models, providing an important reference framework for both the experimental design and interpretation of the present findings.

In addition, differences in animal age and sex between this study and earlier work from our research group may partially limit inter‐study comparability. Future studies should incorporate both thermoneutral and heat‐stress conditions within the same animal population, together with antioxidant supplementation and direct meat quality phenotyping, and integrate molecular‐level analyses to more comprehensively elucidate the mechanisms by which plant‐derived antioxidants improve production performance and meat quality in ruminants.

## 5 Conclusions

Based on multidimensional analyses, including growth performance, physiological parameters, blood indices, and rumen microbiota, the following conclusions were drawn: Mog V (1200 mg/kg) exhibited the most pronounced overall effect, significantly increasing ADG and feed intake, reducing the FCR, improving RR and RT, and optimizing energy metabolism through the enrichment of *Firmicutes* and *Succiniclasticum*. EGCG (800 mg/kg) was particularly effective in enhancing SOD activity, suppressing TNF‐α, and regulating acetate metabolism. RES (150 mg/kg) markedly reduced MDA levels, enriched *Verrucomicrobia* and *Fibrobacteres*, and elevated concentrations of propionate, butyrate, and other SCFAs. All three extracts effectively modulated HS‐related indicators by decreasing serum HSP70, HSP90, and pro‐inflammatory cytokines (TNF‐α, IL‐1β, IL‐6), while increasing the activities of antioxidant enzymes (GSH‐Px, CAT, T‐AOC). Collectively, Mog V demonstrated the greatest potential in alleviating HS and promoting healthy growth in Hu sheep, providing both theoretical evidence and practical reference for sustainable livestock production.

## Author Contributions

**Yirong Wei:** writing – original draft, visualization, methodology, investigation, formal analysis, data curation, conceptualization. **Jun Lu:** writing – original draft, visualization, methodology, investigation, formal analysis, data curation, conceptualization. **Shaoqiang Wu:** investigation, formal analysis. **Zhihua Mo:** investigation, methodology. **Haien He:** investigation, methodology. **Yulong Shen:** investigation, methodology. **Jianwei Zou:** investigation, methodology. **Cheng Xing:** investigation, methodology. **Yanna Huang:** writing – review and editing, supervision, resources, methodology, investigation, data curation, conceptualization. **Qinyang Jiang:** writing – review and editing, supervision, resources, methodology, investigation, data curation, conceptualization.

## Funding

This research was funded by the Key Research and Development Program of Guangxi (no. Guike AB23026081), the National Modern Agricultural Industry Technology System Guangxi Innovation Team (nycytxgxcxtd‐2021‐09).

## Conflicts of Interest

The authors declare no conflicts of interest.

## Supporting information


**Table S1:** Effects of different additives on rumen microbial α‐diversity indices in Hu sheep.

## Data Availability

Data available on request from the authors.
