# Supplementary material for: Comparative Analysis of the Effects of Mogroside V, Epigallocatechin Gallate, and Resveratrol on Growth Performance, Blood Parameters, Rumen Microbiota, and Short‐Chain Fatty Acid Metabolism in Heat‐Stressed Hu Sheep
Source: Food Sci Nutr. 2026 Jan 15;14(1):e71455. doi: 10.1002/fsn3.71455 (PMC12808817; doi:10.1002/fsn3.71455)
Supplement: Supplementary file 1 — Table S1: Effects of different additives on rumen microbial α‐diversity indices in Hu sheep. [file FSN3-14-e71455-s001.docx]

# Supplementary Material

**Supplementary Table 1.** Effects of Different Additives on Rumen Microbial α-Diversity Indices in Hu Sheep.

| **Item** | **C** | **V** | **E** | **R** |
| --- | --- | --- | --- | --- |
| Chao1 | 3925.56±104.98^a^ | 3645.06±143.04^ab^ | 3291.5±177.53^bc^ | 3027±281.5^c^ |
| Observed_species | 3589.58±104.92^a^ | 3338.5±150.02^ab^ | 3045.02±168.66^bc^ | 2790.64±241.44^c^ |
| Shannon | 9.99±0.12^a^ | 9.95±0.15^a^ | 9.61±0.18^a^ | 9.64±0.23^a^ |
| Simpson | 1 | 1 | 0.9 | 0.99 |

Note: Data in the same industry with different lowercase letters indicate significant differences (*P*<0.05), while data with the same lowercase letters indicate no significant differences (*P*>0.05).
